# Multifocal Small Bowel Gastrointestinal Stromal Tumor (GIST): A Case Report

**DOI:** 10.7759/cureus.70678

**Published:** 2024-10-02

**Authors:** Noah Jemedafe, Zaka U Jan, Dominic Ayegba, Aboje Adugba, Mohammed Y Jan

**Affiliations:** 1 Surgical Oncology, Sultan Qaboos Comprehensive Cancer Care and Research Centre (SQCCCRC), Muscat, OMN

**Keywords:** gastrointestinal stromal tumour (gist), mesenchymal, multifocal, small bowel, spindle cell

## Abstract

Gastrointestinal stromal tumours, also known as GIST, are a rare subset of soft tissue sarcomas. They are the most common mesenchymal neoplasms of the GI tract and can be found multifocal intra-operatively.

We present a 44-year-old male patient who presented with vague upper abdominal pain of one-week duration associated with non-bilious vomiting. His CT abdomen with IV contrast revealed a non-enhancing intraperitoneal mass, which displaced the small bowel and mesentery; however, there was no obstruction or strangulation. A CT-guided biopsy was done and showed a low-grade gastrointestinal stromal tumor (GIST). Interestingly, during diagnostic laparoscopy, in addition to the large serosal ileal mass, he had multiple smaller lesions throughout the small bowel. He underwent resection of all the masses. He made an excellent recovery and was discharged home on postoperative day three. Small bowel GIST can rarely present as multiple lesions. Examination of all bowels during surgery is mandatory.

## Introduction

Gastrointestinal stromal tumours (GISTs) are peculiar as they have a variable malignant potential ranging from indolent tumours to rapidly progressive cancers [1].

GISTs arise from the connective tissue outer lining of the gastrointestinal (GI) tract and can occur anywhere along its length. They are the most common mesenchymal tumors of the gastrointestinal (GI) tract. The most common site is the stomach (50-60%), followed by the small intestine (30-40%) and the colon (5-10%) [2]. It is interesting to note that GIST may rarely arise from other non-GI sources, such as the omentum, mesentery, and retroperitoneum and is reported at around 5.5% [3]. They may present as a solitary or multifocal mass.

GIST has an incidence of 10-20 per million and an estimated malignancy rate of 20-30%; however, the data source needs review as the diagnostic criteria for GIST have been recently amended [4]. Aggressive features are less seen in gastric GIST compared to tumours from other sources [5]. The age range is around 10-100 years with a median age of mid-60s and tends to occur equally between male and female patients [3]. Around 80% of GIST have varying molecular changes, predominantly mutually exclusive activating KIT or platelet-derived growth factor receptor alpha (PDGFRA) mutations, but other rare subtypes also exist [6].

They are mesenchymal neoplasms showing differentiation toward the interstitial cells of Cajal and are typically characterized by the expression of the receptor tyrosine kinase KIT (CD117).

Most patients typically present with nonspecific abdominal symptoms such as pain, discomfort, and fullness. If the tumor is large enough, there may be a palpable mass, and these tumours can cause bowel obstruction, perforation, and bleeding. Other patients are discovered incidentally during workup for other conditions [7].

## Case presentation

A 44-year-old gentleman with no previous medical history was referred from a secondary health facility. He was complaining of upper abdominal pain of one-week duration that progressively worsened in severity, associated with multiple episodes of recurrent, non-bilious vomiting. No melena or other gastrointestinal symptoms, no fever. He had no history of abdominal surgery.

Clinical examination of his abdomen revealed a mildly distended abdomen, soft, non-tender, with no palpable mass. Bowel sounds were audible and normoactive. Digital rectal examination was unremarkable.

His laboratory test showed a total white blood cell count of 9.81 X 10*3, hemoglobin of 14.7g/dL, and slightly raised C-reactive protein of 8.77. His liver function test showed a raised alanine transaminase (ALT) of 103 umol/L, with a normal renal function test, as shown in Table 1.

**Table 1:** Showing the lab values WCC - white cell count; ALT - alanine transaminase; AST - aspartate aminotransferase; ALP - alkaline phosphatase; BUN - blood urea nitrogen

Lab tests	Result	Reference value
Hb	14.7g / dL	11.50-15.50g/dL
WCC	9.81 x 10*3/uL	2.20-10.00 x10*3/uL
Platelets	357	150-450 x10*3/uL
C-reactive protein	8.77	0-5 mg/L
ALT	103	0-41 U/L
AST	17	0-40 U/L
ALP	106	40-129 U/L
Urea (BUN)	4.7	2.8-8.1mmol/L
Creatinine	72	59-104 umol/L

**Figure 1:**
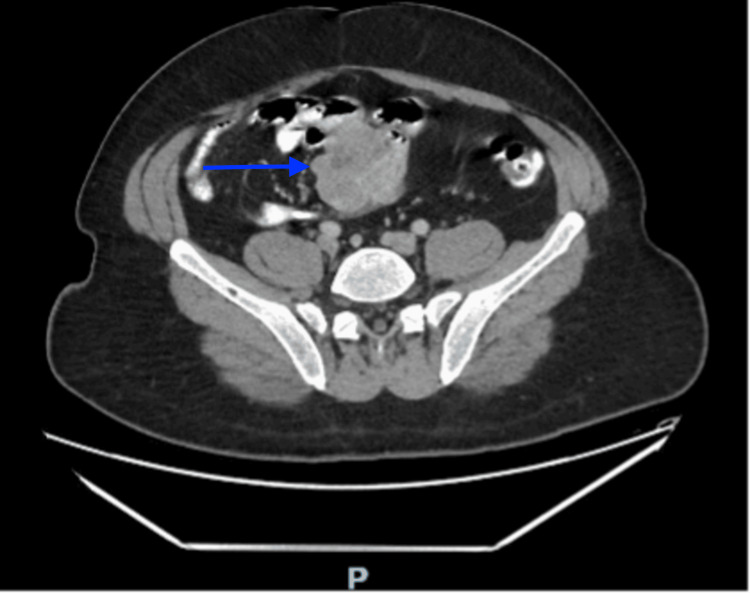
Axial view showing the heterogeneously enhancing mass (blue arrow) displacing the adjacent small bowel loops

**Figure 2:**
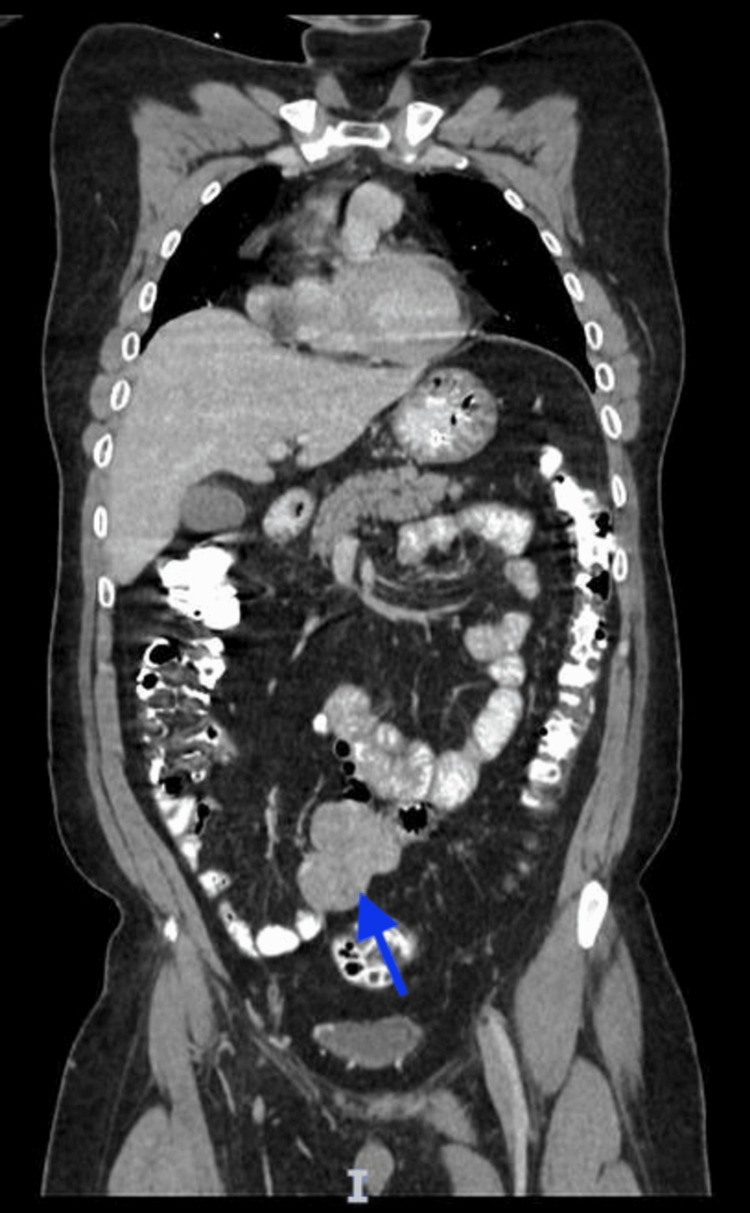
Coronal view showing the heterogeneously enhancing mass (blue arrow) displacing the adjacent small bowel loops

A CT scan of the abdomen and pelvis showed an intraperitoneal heterogeneous enhancing mass in the central small bowel mesentery region that measured 54 x 56 x 68 mm with two foci of calcification. The mass displaced the adjacent small bowel loops with no sign of strangulation or obstruction (Figures 1, 2).

**Figure 3:**
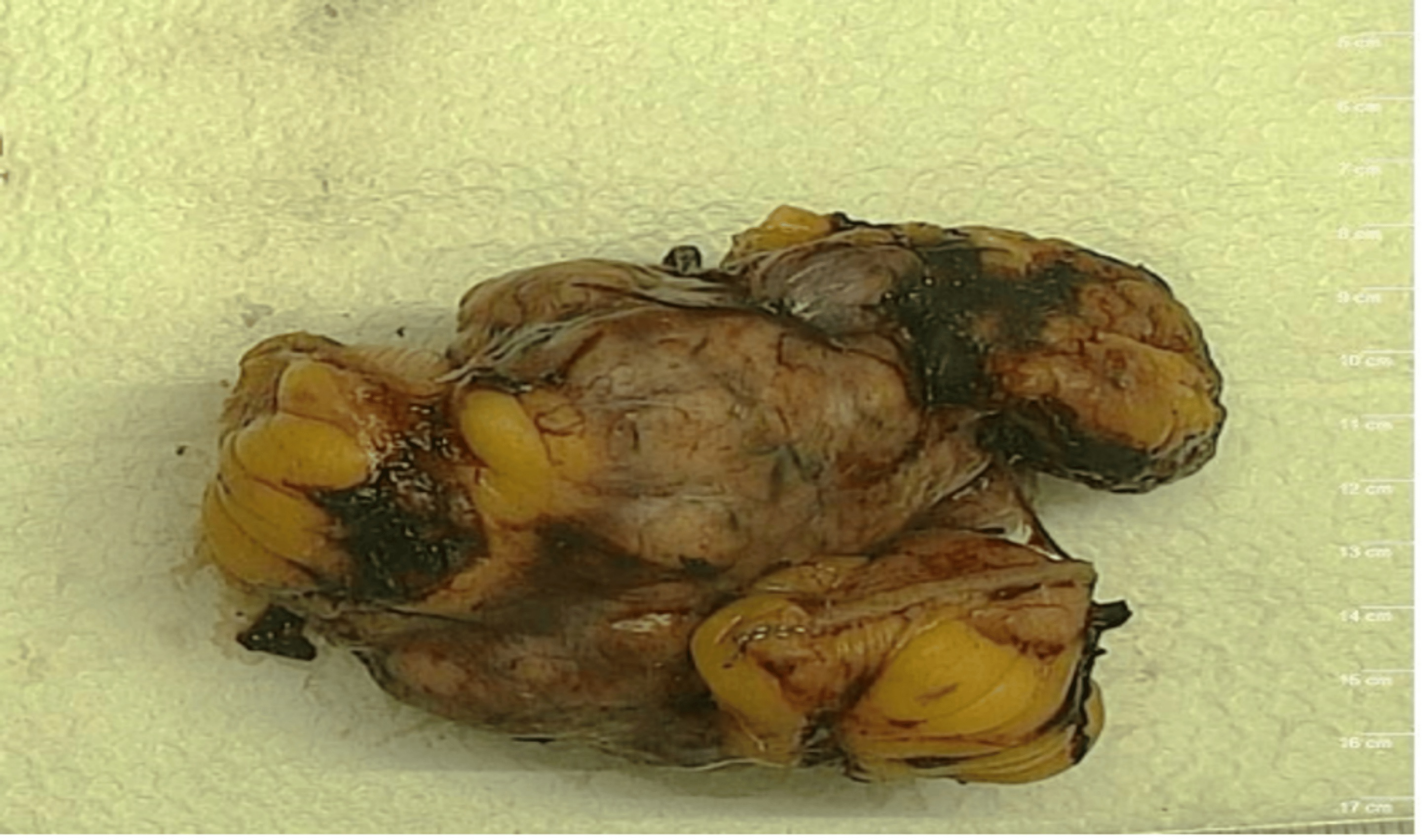
Gross specimen - a segment of small intestine with a nodular mass

An image-guided biopsy of the mass showed a low-grade gastrointestinal stromal tumor (GIST), spindle cell type. The patient underwent a diagnostic laparoscopy with intraoperative findings of a soft tissue mass originating from a small bowel wall (ileum) measuring 6.5 x 5.5 x 3.5cm. Smaller similar masses were found away from this mass involving the small bowel wall at different sites at the duodenal-jejunal junction. He had en-bloc resection of the mass with the intervening loops of the bowel and excision of multiple nodules on other bowel segments followed by two side-to-side ileo-ileal anastomoses (Figure 3).

**Figure 4:**
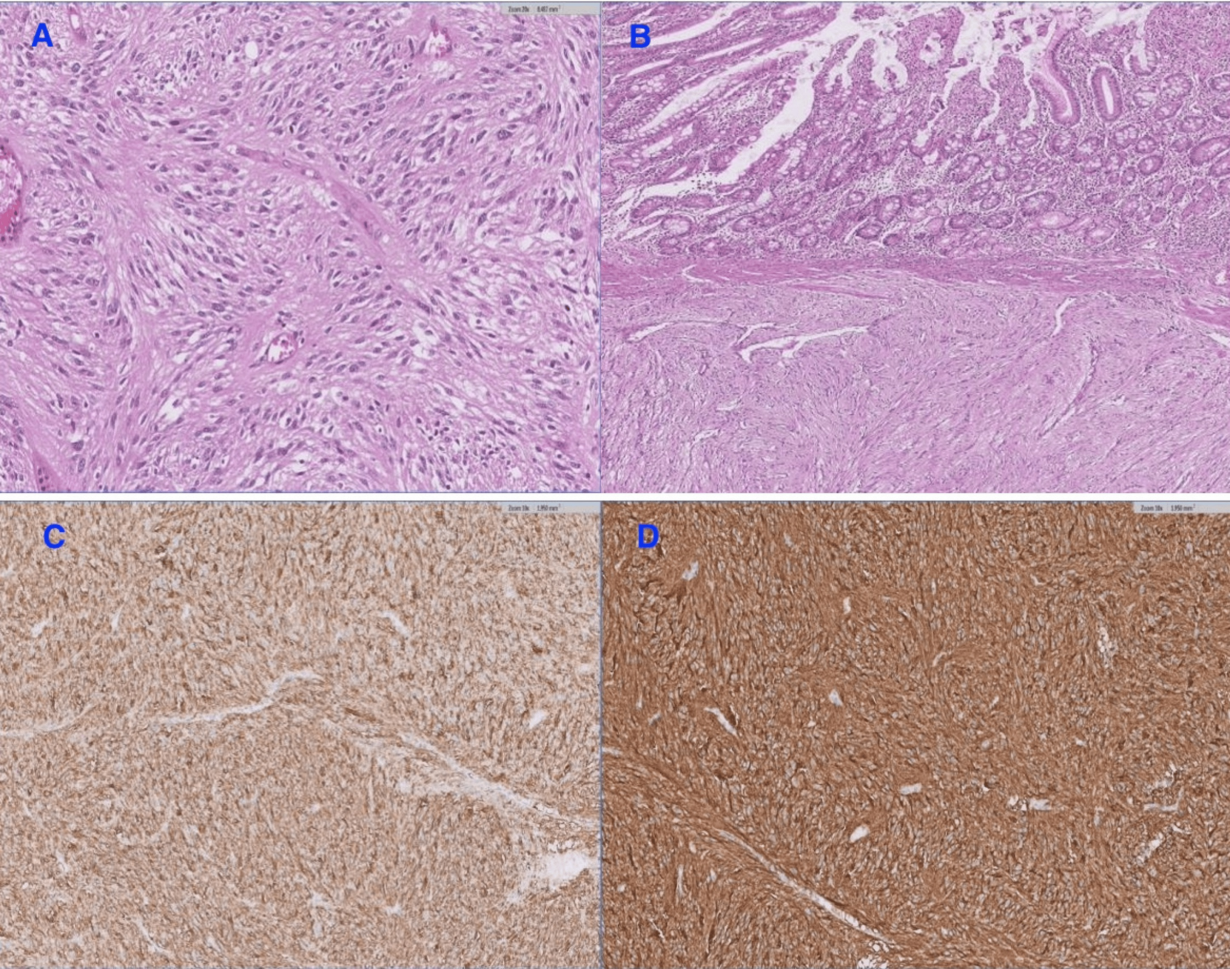
Histopathological findings A, B) Small bowel with a submucosal spindle cell tumor (A) showing fascicles and low grade atypia (B), H&E 20x; C, D) Tumor cells are positive for CD117 (C) and DOG-1 (D), immunohistochemistry  (IHC) 20x .

Histopathology of the specimen revealed a mass measuring 6.5 x 5.5 x 3.5 cm between two loops of the small intestine with areas of hyalinization and hemorrhage. Microscopy showed a well-circumscribed spindle cell tumor with variable cellularity (low and high). Mitoses are up to 3/50 high power field (HPF; <5/5mm2). The immunohistochemistry was positive: CD34, CD117, and DOG1. The diagnosis favored gastrointestinal stromal tumor, spindle cell type, low-grade (mitoses <5/5mm2). The risk stratification was a moderate risk. All other specimens showed similar pathology. All the margins were negative for a tumor, and the resection margins were clear (Figure 4).

He recovered well post-operatively and was discharged in stable condition. His case was discussed in a multi-disciplinary team (MDT) meeting, and considering his moderate risk, he was commenced on adjuvant treatment with imatinib for three years. He is currently on month four of follow-up while on imatinib.

## Discussion

Overall, gastrointestinal stromal tumors are rare tumors that develop from the GI mesenchymal tissue arising because of genetic mutation. It can occur anywhere along the GI tract; thus, the symptoms may vary. It may pose a diagnostic dilemma or mimic other surgical conditions. It can present both as solitary or multifocal lesions, with the latter associated with a worse prognosis. A multifocal lesion may be part of a syndrome such as neurofibromatosis or Carney's triad. It may reflect metastatic disease; however, this is unlikely in our patient, whose tumor biology showed low-grade features,

Life-threatening complications, which range from bowel obstruction, bleeding (5-10%) [8] - although acute massive GI bleeding related to small intestine GIST is unusual [9] - perforation to local invasion with metastases may occur, especially in aggressive malignant variants if undetected earlier. Thus, a high index of suspicion, along with knowledge of the nature and presentation of GISTs, is crucial.

Diagnosis is usually possible with a CT scan. A preoperative biopsy is not necessary when the lesion is considered suspicious of GIST and is surgically resectable. However, a biopsy of the tumor is indicated if, radiologically, the diagnosis is in doubt and/or in the case of locally advanced tumors requiring neoadjuvant therapy [10]. In our case, the patient was initially diagnosed using a non-contrast CT with subsequent CT-guided biopsy in the same setting.

Treatment of GIST includes assessment of the extent and progression of disease in addition to appropriate risk stratification. Surgery is the mainstay of treatment in over 70% of patients with primary localized GIST. Using the latest advances in laparoscopic and robotic surgery techniques is useful in many cases but is limited by location and size. Lymphadenectomy is rarely performed as metastasis to lymph nodes is uncommon [11-12].

Neoadjuvant tyrosine kinase inhibitors are indicated for large, unresectable, or metastatic tumors. Novel targeted therapy (ipilimumab, nivolumab, and endoscopic ultrasound alcohol ablation) continues to show promising results. This has led to improved survival rates both for patients with localized GIST and those with advanced disease, although drug resistance tends to occur after 18 months of imatinib therapy. Once clinical progression develops, increased doses of imatinib or sunitinib (a multitarget tyrosine kinase inhibitor) can restore GIST response in some patients, at least temporarily [13].

The prognosis following the resection of a primary, localized GIST is a function of tumor size, location, and mitotic rate [14].

## Conclusions

Multi-focal small bowel GIST is a rare entity of this disease. A pre-operative evaluation may fail to diagnose this. Detailed intra-operative examination of all the bowels is mandatory to exclude this possibility. Surgical resection remains the gold standard, with the need for adjuvant treatment given the high-risk features in these cases.
